# T190. ASSOCIATION OF THE HUMAN MU-OPIOID RECEPTOR GENE POLYMORPHISM ASN40ASP WITH SCHIZOPHRENIA

**DOI:** 10.1093/schbul/sby016.466

**Published:** 2018-04-01

**Authors:** Anna Li, Jiaxin Yuan, Te-An Chen, Meghan Helm, Steve Dubovsky, Junzhe Xu

**Affiliations:** 1 VHAWNY HealthCare System; 2 Medical School, SUNY at Buffalo

## Abstract

**Background:**

In humans, opioidergic neurotransmission appears to modulate a variety of behaviors, including the stress response and cognitive processes, as well as anxiety and psychosis. One neurobiological process which may be modified by the Asn40Asp polymorphism of the μ opioid receptor is the HPA axis response to stress. Hypothalamic corticotropin-releasing hormone (CRH) neurons, which affects glucocorticoid release by stimulating pituitary adrenocorticotropin (ACTH) secretion, are directly and indirectly inhibited by β-endorphin-producing neurons via the μ opioid receptor (OPRM). Both exaggerated and blunted HPA responses to stress have been associated with high risk for psychosis. Many studies have suggested that opioids play an important role in response to stress, motivation, and numerous psychiatric entities. The present association study tested the hypothesis that the Asn40Asp substitution polymorphism confers susceptibility to schizophrenia.

**Methods:**

After informed consent was obtained, 100 schizophrenia patients and 100 control subjects were enrolled in this study. Genomic DNAs were extracted from peripheral blood by using the modified SDS/Proteinase K procedure. The genotypes of the Asn40Asp polymorphism of the μ opioid receptor were assessed by allele-specific polymerase - chain reaction. The PCR products were digested by restricted enzyme.

**Results:**

The frequency of the Asp40 allele was significantly increased in all schizophrenia patients (Fisher’s Exact Test P= 0.0118). There were no associations the Asn40Asp polymorphism of the μ opioid receptor with substance dependence among schizophrenia patients and normal control.

**Discussion:**

The opioidergic neurotransmitter system plays an important role in regulating activation of the hypothalamic-pituitary-adrenal (HPA) axis. Initial activation of the HPA axis occurs at the level of the paraventricular nucleus of the hypothalamus, where neurons that produce corticotropin releasing factor (CRF) are located [Bell et al., 1998]. CRF neurons in this area express μ-opioid receptors and are under tonic inhibition by neurons of the arcuate nucleus that contains β-endorphin [Wand et al., 1998]. Genetic factors appear to be important modulators of HPA axis activation. The HPA axis appears to be involved, including the normal stress response [Bond et al., 1998; LaForge et al., 2000] and psychosis in which HPA axis dynamics appear to be abnormal. Similarly, there is growing evidence that altered opioidergic neurotransmission and HPA axis dynamics may affect alcohol- and drug-seeking behaviors [Piazza and Le Moal, 1997; Kreek and Koob, 1998].

